# Thoracic spinous process nonunion as an unusual cause of back pain: a case report and review of the literature

**DOI:** 10.1186/s13256-023-04109-3

**Published:** 2024-01-03

**Authors:** Gilles Dietrich, Raphaël Richard, Alain Akiki, Sebastien Levy, Benoit Maeder

**Affiliations:** 1 Orthopaedic Surgery and Traumatology Department, Lausanne University Hospital and University of Lausanne, Lausanne, Switzerland; 2 Orthopaedic Surgery and Traumatology Department, Riviera-Chablais Hospital, Rennaz, Switzerland; 3 Radiology Department, Riviera-Chablais Hospital, Rennaz, Switzerland

**Keywords:** Spinous process, Nonunion, Pseudoarthrosis, Resection, Case report

## Abstract

**Background:**

Purely isolated spinous processes fractures are rare and are usually treated conservatively, although a few authors have reported cases of nonunion that ultimately required surgical resection.

**Case presentation:**

We present a case of an isolated T6 spinous process pseudoarthrosis that was treated by surgical resection of the tip of the spinous process. A 34-year-old Caucasian male patient was complaining of mid-thoracic back pain without neurologic impairment more than 2 years after an isolated spinous process fracture. Magnetic Resonance Imaging (MRI) and Single Photon Emission Computed Tomography (SPECT) revealed a nonunion. We performed a resection without further complication.

**Conclusion:**

Although spinous process nonunions may in some cases be well tolerated, surgical resection appears to be a reliable option in case of persistent symptoms. This illustrated case shows the description of an isolated thoracic spinous process nonunion and its surgical treatment.

## Introduction

Isolated spinous process (SP) fractures are often described in the cervico-thoracic junction [1, 2]. These fractures may result from direct blows to the SP, avulsion of SP during forced flexion, impaction during hyperextension or fatigue fracture arising from repetitive tensile force [2]. Fatigue fracture of SP of the cervico-thoracic junction is known as the “clay shoveler’s fractures” [3].

SP lesions are often associated with injuries to the posterior ligamentous complex and fracture stability should be carefully assessed in such cases [4]. If the fracture is isolated, conservative treatment consisting of pain management eventually combined with physical therapy is largely advocated and surgery is rarely needed [2]. Good results are also reported with conservative treatment in the setting of multiple contiguous SP fractures [5, 6].

In some cases, persistent pain following isolated SP fractures is related to the development of a SP nonunion [7]. A very limited number of authors have previously described surgical resection of symptomatic SP nonunions and only one author has described it at the thoracic level [9–12]. Moreover, these were three athletic skeletally immature patients.

The purpose of this case report and review of the literature was to describe the results of treatment of a patient with symptomatic pseudarthrosis of the mid-thoracic SP who failed conservative treatment. The information in this report should be a useful reference for physicians treating these injuries.

The following case is presented in accordance with the CARE Guidelines.

Written informed consent was obtained from the patient for publication of this case report and any accompanying images. A copy of the written consent is available for review by the Editor-in-Chief of this journal.

## Case

A 34-year-old Caucasian man, cook, smoker, but with no prior medical or surgical history, presented to the Emergency Department with explicit, acute-onset, left lower thigh pain and a midline thoracic pain after sustaining a rear-end motor vehicle accident while in the front car. A total body computed tomography (CT) scan showed a left femoral shaft fracture and an isolated T6 SP fracture. Intramedullary nailing of the femur was performed the same day and conservative treatment was favored for the T6 SP fracture. Three months after the accident the patient started to complain of mild upper back pain. As time went by, left lower limb function returned to normal while the upper back pain became increasingly bothersome. The patient’s description of the pain was mechanical since it intensified with activity.

Physical examination after 6 months of follow-up revealed distinct tenderness with minimal swelling on palpation of the mid-thoracic spine. Discrete limitation of range of motion of the spine was noted and pain was present at the same level during trunk flexion or hyperextension. No neurological compromise was present. Pain management and physical therapy with spinal rehabilitation programmes were conducted with moderate benefit on symptoms through 18 months of follow-up.

**Fig. 1:**
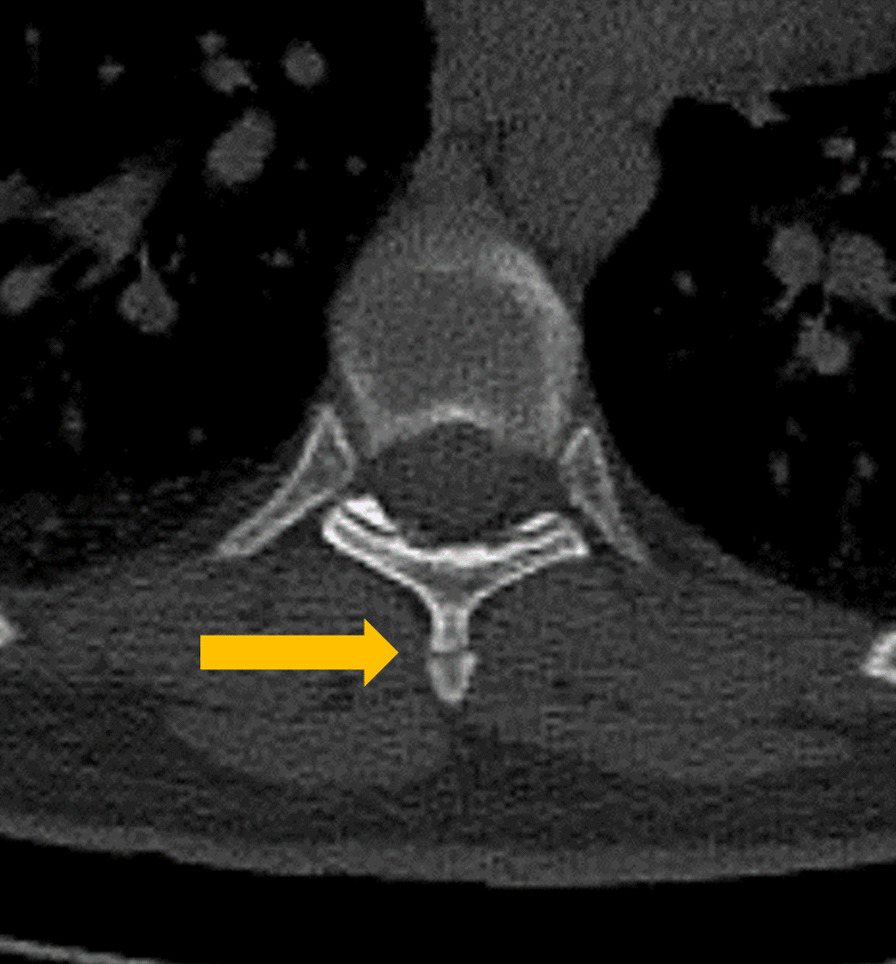
Computed tomography axial view showing a T6 spinous process nonunion (gold arrow) 2 years after the fracture

**Fig. 2:**
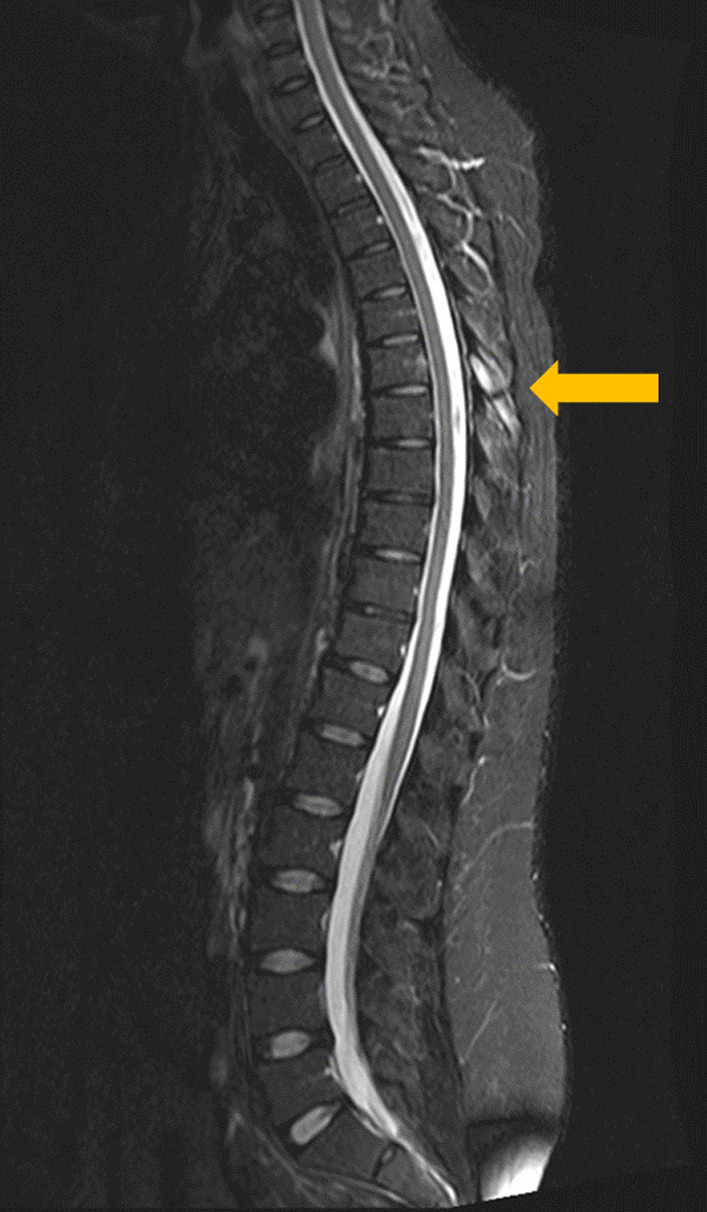
Short tau inversion recovery sequence magnetic resonance imaging sagittal view showing an isolated T6 nonunion (gold arrow) 2 years after the fracture

**Fig. 3:**
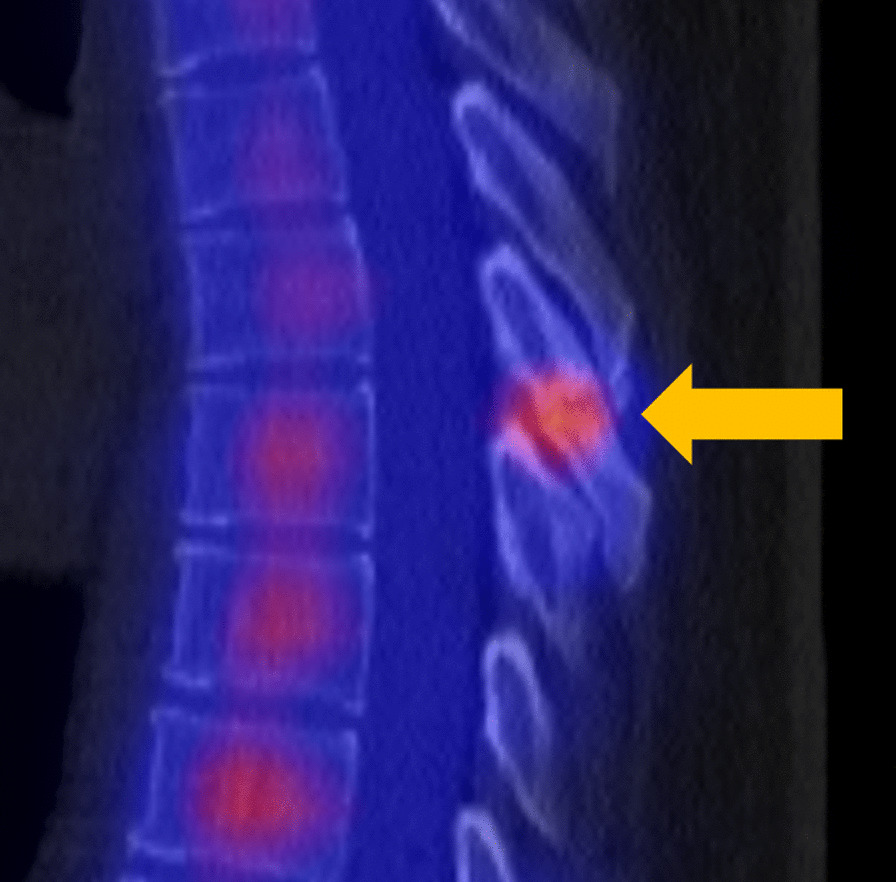
Single Photon Emission Computed Tomography showing increased signal uptake at the location of T6 spinous process nonunion (gold arrow) 2 years after the fracture

More than 2 years after the SP fracture, Single Photon Emission Computed Tomography (SPECT) combined with Magnetic Resonance Imaging (MRI) showed a pseudoarthrosis of T6 SP and no other identifiable cause of pain (Figs. 1, 2, 3).

**Fig. 4:**
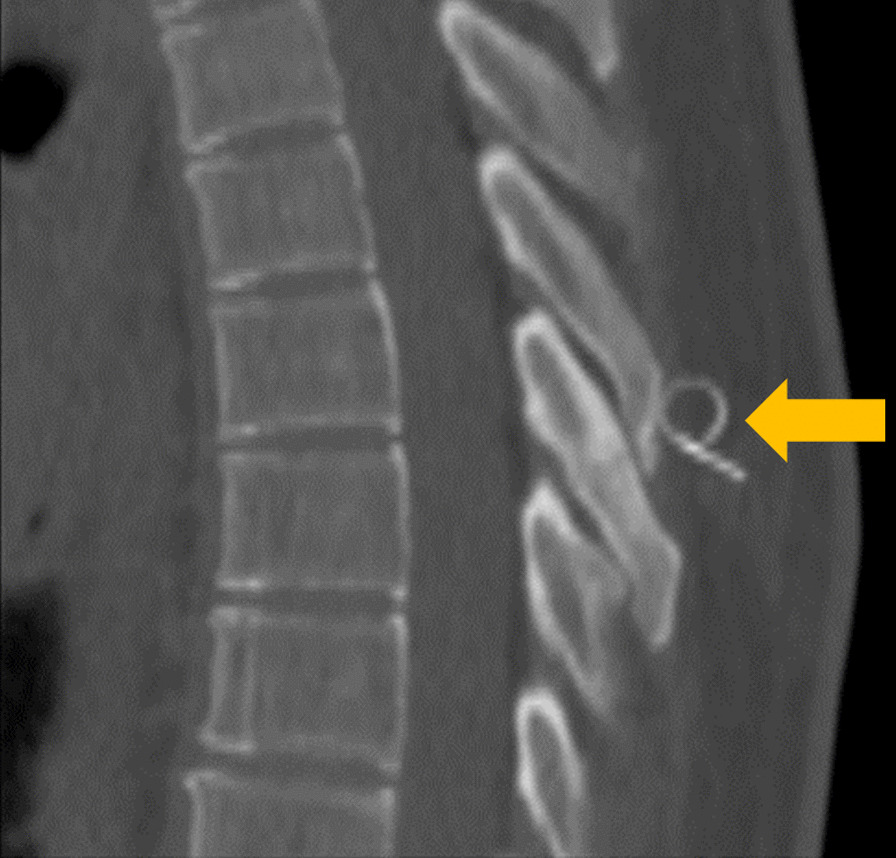
Pre-operative computed tomography with a landmark wire (gold arrow) to ensure the correct vertebral level

A guided steroid injection was performed, which alleviated the pain significantly for a short period. Because of persistent pain, evaluated at 8/10 on the visual analog scale (VAS), surgical resection of the T6 SP was discussed and accepted by the patient twenty-eight months after the accident. Prior to the surgery a landmark wire was placed under CT to confirm the vertebral level (Fig. 4). Intra-operatively, after a 3 cm midline incision we removed the tip of the T6 SP.

The postoperative course was uncomplicated. The nonunion was confirmed after histological examination of the fragment, which concluded that reactive fibrocartilage was present. After 6 weeks the patient was able to return to work and his usual daily activities without any limitation. Six months after surgery he did not complain of any limitation and reported intermittent mild upper back pain ranging from 0/10 to 2/10 on the VAS. Clinical examination revealed no pain on palpation and a full, painless range of motion of the trunk.

## Review of literature

A narrative literature research was performed in MEDLINE (PubMed) database. The keywords used were spinous process, nonunion, pseudoarthrosis, surgical treatment, resection. Only papers published in English were considered. Surgical resection of SP nonunion are only described in few case reports or minor case series.

## Discussion

To our knowledge, no previous patient case of an isolated thoracic SP fracture in an adult has been reported.

Isolated SP fractures are generally treated conservatively. Most patients experience favorable outcome, but in some cases, persistent pain could be related to SP nonunion. Pseudoarthrosis can probably be well tolerated, with no or limited symptoms, as described by Kose *et al.* [7]. An unknown proportion of SP nonunions are not documented because patients are doing well. However, in some cases, SP nonunions or malunions may be associated with chronic pain or difficulty with certain activities such as heavy work or sports [8].

In our case, pseudarthrosis of a thoracic SP was suspected to be the primary source of symptoms. Mild strains were probably present when the patient was active, as he described mechanical-looking pain that intensified with activity. This was underscored by a clinical examination showing tenderness to palpation and localized pain in the absence of neurological impairment together with radiological studies (SPECT combined with MRI) ruled out other possible causes of upper back pain and confirmed the presence of a nonunited SP.

Various studies have described the surgical treatment of SP pseudoarthrosis. Table 1 highlights the treatment and the outcome reported in these studies. A large proportion of the patients who were ultimately treated with surgical resection were young athletes with persistent pain who were unable to return to sports [9, 10]. Murphy *et al.* described the cases of three adolescents who underwent surgical resection of an upper thoracic SP pseudoarthrosis. All three lesions were located at T1, and patients were treated an average of ten months after the initial injury [9]. Fayyazi *et al.* described the case of a 16 year old basketball player with a symptomatic nonunion of a lumbar SP who was treated with surgical excision after conservative treatment failed [10]. Hirsh *et al.* described a pseudoarthrosis of C2 SP that was treated by surgical resection [11]. Brown *et al.* reported an isolated fracture of C7 SP that was treated by excision after conservative treatment failed [12].

**Table 1:** Reports of spinous process nonunion resection

Authors	Patient age (years)	Level	Clinical presentation	Duration of symptoms prior to surgery (months)	Type of surgery	Outcome and complications
Brown	38	C7	Pain	3	SP resection	No pain, no complication
Hirsch	29	C2	Pain	8	SP resection	Full RTW, no pain, no complication
Fayyazi	16	L3	Pain	24	SP resection	Full RTS, no pain, no complication
Murphy	Mean: 14 (3 patients)	T1	Pain	Mean: 10	SP resection	Full RTS, no pain, no complication

*SP* spinous process, *RTW* return to work, *RTS* return to sport

Isolated SP fractures are most common at the level of T1, followed by C7, T2, T3 and C6 [13]. The cervicothoracic junction may be at greater risk because of the difference in mobility between the mobile subaxial cervical spine and the more rigid thoracic spine.

All available studies describing patients who were ultimately treated by surgical resection of a SP pseudoarthrosis report a good outcome without complications. We believe that surgical excision of the spinous process is a reliable and safe option if back pain is suspected to be related to SP pseudoarthrosis and persists after conservative management has failed. We recommend performing a guided steroid injection prior to resection, as it may be a good prognostic indicator if the pain decreases.

## Conclusion

When conservative treatment of an isolated spinous process nonunion has failed and other causes of upper back pain have been ruled out, nonunion resection is a reliable surgical option with limited risks.

## Data Availability

The data used to support the findings of this study are included within the article.

## Abbreviations

SP: Spinous process
CT: Computed tomography
SPECT: Single photon emission computed tomography
MRI: Magnetic resonance imaging
VAS: Visual analog scale
